# Persistence and Dissemination of Enrofloxacin and Ciprofloxacin Residues: The Hidden Role of Litter and Droppings in the Emergence of Antimicrobial Resistance

**DOI:** 10.3390/ani16020333

**Published:** 2026-01-21

**Authors:** María Belén Vargas, Camila Nettle, Ignacia Soto, Ekaterina Pokrant, Aldo Maddaleno, Lisette Lapierre, Javiera Cornejo

**Affiliations:** 1 Laboratory of Food Safety, Department of Preventive Animal Medicine, Faculty of Veterinary and Animal Sciences, University of Chile, Santiago 8820808, Chile; maria.vargas.s@ug.uchile.cl (M.B.V.); camila.rojas.n@ug.uchile.cl (C.N.); ignacia.soto@ug.uchile.cl (I.S.); katiavalerievna@uchile.cl (E.P.); 2 Doctorate Program of Forestry, Agricultural and Veterinary Sciences (DCSAV), Southern Campus, University of Chile, Santa Rosa 11735, La Pintana, Santiago 8820808, Chile; 3 Laboratory of Veterinary Pharmacology (FARMAVET), Faculty of Veterinary and Animal Sciences, University of Chile, Santiago 8820808, Chile; amaddaleno@veterinaria.uchile.cl; 4 Laboratory for the Diagnosis of Bacterial Pathogens and Antimicrobial Resistance, Department of Preventive Animal Medicine, Faculty of Veterinary and Animal Sciences, University of Chile, Santiago 8820808, Chile; llapierre@uchile.cl

**Keywords:** antimicrobial resistance, ciprofloxacin, enrofloxacin, *Escherichia coli*, poultry, residues

## Abstract

The objective of this study was to evaluate the persistence and environmental dissemination of enrofloxacin (EFX) and its metabolite ciprofloxacin (CFX) residues in poultry systems, as well as their effect on antimicrobial resistance in *Escherichia coli*. The experimental design included three groups: one treated group (A) and two untreated groups (B.1 and B.2), located in pens adjacent to or within 30 cm of group A to assess residue transfer. Birds in group A received EFX orally (10 mg·kg^−1^ every 24 h for 5 days) via orogastric tube. EFX and CFX residues persisted in litter and excreta for up to 18 days post-treatment, reaching initial concentrations of 20,968 µg·kg^−1^ in litter and 884.8 µg·kg^−1^ in droppings in group A. Significant differences were detected between groups (Kruskal–Wallis, *p* < 0.05), confirming greater accumulation in the treated group and environmental dissemination. *E. coli* isolates obtained from litter and droppings from group A showed 73% resistance after treatment and correlated positively with residue concentration (ρ = 0.53). While the untreated groups B.1 and B.2 showed resistance rates of 24% and 13%, respectively, the control group exhibited a resistance rate of 3.3%. This study shows the detection of low levels of EXF and CFX residues in the litter of untreated groups, indicating limited dispersion. These findings highlight the importance of proper byproduct management and targeted environmental monitoring within the One Health framework, as continuous environmental exposure over time, combined with the persistence of this compound, may contribute to the selection of resistant bacteria.

## Simple Summary

Enrofloxacin is a widely used antimicrobial agent for treating poultry diseases. However, the presence of its residues is a significant concern due to their introduction into the production environment and the selection of quinolone-resistant pathogens, with litter and droppings from birds being a potential source of contamination as there are no regulations establishing maximum residue limits in these matrices. This study aimed to evaluate litter and droppings as reservoirs of enrofloxacin and ciprofloxacin residues and their relationship to the development of antimicrobial resistance in *Escherichia coli* in a controlled poultry production model. Following enrofloxacin treatment (10 mg·kg^−1^ every 24 h for 5 days), high concentrations of residues were detected in the litter and droppings of treated birds exceeding the minimal inhibitory concentration of *E. coli* to enrofloxacin, reaching elevated levels in feces by day 3 post-treatment and remaining above the limit of quantification until day 18. Ciprofloxacin residues persisted in feces until day 12 and were still detectable in litter on day 18. The presence of residues in untreated groups suggests environmental dissemination. Regression analysis showed a significant positive relationship between residue concentration and resistance rate. These findings highlight the potential of poultry litter and droppings as reservoirs and vectors of antimicrobial residues and resistance, demonstrating the persistence and dissemination of residues in the production environment, as well as their impact on *E. coli* resistance. This underscores the importance of proper waste management and mitigating its negative impact from a One Health perspective.

## 1. Introduction

The sustained growth of intensive animal production systems has driven the widespread use of antimicrobials, which are widely used for disease prevention and control to reduce animal morbidity and mortality [1,2,3]. It is estimated that approximately 73% of antimicrobials marketed worldwide are intended for use in livestock [1], where poultry farming is a significant consumer. Projections indicate an 11.5% increase in antimicrobial use in animals intended for human consumption by 2030 [4]. Indiscriminate use has contributed to the emergence and spread of antimicrobial resistance (AMR), which is considered one of the greatest threats to global public health [5,6]. Without effective interventions, AMR could result in nearly 10 million deaths annually by 2050 [6].

In poultry production, antibiotics are used for therapeutic, prophylactic, and metaphylactic purposes, ensuring the health of birds and the profitability of production systems [1,7]. The resulting antimicrobial residues are considered emerging contaminants of global relevance, as they can persist in animal products and by-products. Therefore, international organizations have established maximum residual limits (MRLs) to prevent consumer exposure [8,9].

Despite this, inedible by-products, such as poultry litter, are not subject to direct regulation. This material—composed of droppings, feed scraps, feathers, and litter—can accumulate excreted antimicrobials in proportions ranging from 17% to 90%, either in their original form or as active metabolites [10,11,12]. While most antibiotics degrade relatively easily in the environment, certain compounds such as fluoroquinolones have been shown to persist in the environment for more than 120 days. Furthermore, the adsorption of fluoroquinolones to organic substrates is mainly driven by pH, hydrogen bond and electrostatic/cation exchange, resulting in a high adsorption coefficient (Kd/Koc) [13]. Several studies have shown the presence of antimicrobial residues in poultry droppings and litter. Massé et al. [14] detected elevated concentrations of tetracyclines, fluoroquinolones, and sulfonamides; while Pokrant et al. [15] identified residues of florfenicol, tylosin, enrofloxacin, and ciprofloxacin up to 18 days post-treatment. Furthermore, Yévenes et al. [16] reported significant concentrations of enrofloxacin and oxytetracycline even in untreated poultry litter, highlighting the possibility of cross-contamination. These findings highlighted the importance of poultry litter as a potential source of residue contamination in soil and water, as well as a potential risk to animal health as poultry litter is frequently used as fertilizer or, in some cases, as an ingredient in animal feed due to its high nutrient contents. These practices facilitate the reintroduction of antimicrobial residues into the food chain and dispersion into the environment [14,17,18].

Pokrant et al. [15] reported the dissemination of oxytetracycline, with trace concentrations detected in untreated animals. Similar results were obtained for sulfachloropyridazine and tylosin, antimicrobials that persist widely in organic matter, such as chicken litter and feces, and are disseminated during production [19,20]. Therefore, the existence of antimicrobial residues is a cause for great concern due to release into the environment and contribution to the development of pathogens resistant to these drugs [19,21].

Enrofloxacin (EFX), a member of the fluoroquinolone group, is one of the most valuable antimicrobials for veterinary medicine. According to the latest report from the World Organization for Animal Health (WHO), the use of fluoroquinolones accounts for 3.4% of all antimicrobials used in animal health worldwide [6]. This subfamily of quinolones, which is part of a broader family of synthetic antibacterial drugs, is widely used for treating *E. coli* infections in birds [22,23]. The metabolic transformation of EFX occurs in the liver, where up to 10% of this antibiotic is metabolized to ciprofloxacin (CFX), which is an active metabolite obtained by the distillation of the ethyl group from the piperazine ring [24]. This mechanism of action is based on the inhibition of DNA gyrase and topoisomerase IV, essential enzymes for bacterial replication and transcription [22].

EFX has proven to be a molecule that persists in the environment. Swinkels et al. [25] demonstrated that enrofloxacin persists in broiler chicken feces after treatment and remains throughout the production cycle, exceeding the minimum selective concentration (MIC > 0.1 mg kg^−1^). Yang et al. [26] suggest that the persistence of antibiotics in soil fertilized with potentially contaminated manure, modulates the antimicrobial impacts on nitrogen-recycling microbes while causing increases in AMR in the environment [27]. The presence of antibiotic residues in food-producing animal farming systems poses a significant risk to public health, making the control and regulation of these substances essential [28].

The use of fluoroquinolones (FQs) has intensified resistance in foodborne pathogens, posing a significant public health problem. Increased resistance to FQs has been observed in *Salmonella* spp., *Campylobacter* spp., and *E. coli,* and the use of antimicrobials in veterinary medicine is a potential cause [29]. Therefore, it is essential to comprehensively assess the potential sources of contamination of these antimicrobials and the impact on antimicrobial resistance within intensive food animal production systems.

While antimicrobials are a fundamental tool in combating various infectious diseases, use in livestock has played a crucial role in the emergence and spread of antimicrobial resistance [6]. Therefore, the aims of this study are to evaluate the longitudinal persistence of enrofloxacin and ciprofloxacin in poultry litter and droppings after treatment and the role of these matrices as potential environmental drivers of resistance in *E. coli.*

## 2. Materials and Methods

### 2.1. Animal Model and Experimental Conditions

A model of Ross 308 male broiler chickens (Ross, Aviagen Inc., Huntsville, AL, USA) was utilized. The animals were raised in an experimental animal unit under controlled conditions such as temperature (25 ± 5 °C) and relative humidity (50–60%), with free access to feed and purified water. The animal welfare standards approved by the Institutional Committee on Animal Care and Use (CICUA) of the University of Chile were met for the handling, maintenance, and slaughter of the animals, as established by the AVMA “Guidelines for the Euthanasia of Animals” [30] and Directive 2010/63/EU, according to certificate No. 22551-VET-UCH. In addition, biosafety measures described in the “Manual de Normas de Bioseguridad y Riesgos Asociados—Fondecyt—CONICYT” [31] were implemented. This project was granted Certificate No. 177 by the Biosafety Committee of the Faculty of Veterinary and Livestock Sciences at the University of Chile.

The number of birds per pen was calculated following the recommendations of the United States Department of Agriculture’s “Poultry Industry Handbook” [32]. This guideline stipulates a maximum of 34.372 kg·m^2^; therefore, if each adult bird weighs approximately 3.2 kg, then 10 birds would be an appropriate number.

### 2.2. Experimental Design and Pharmacological Treatment

**Figure 1:**
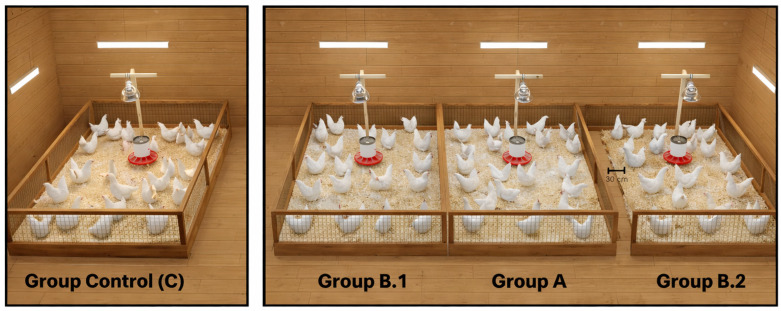
Distribution of the pens in the experimental unit, which has 2 areas, one for the control group and another for the experimental groups.

The experimental design consisted of three experimental groups (A, B.1, and B.2). Group A is the treated group, while groups B.1 and B.2 were untreated animals. Each solid wood pen measured 1 m^2^; it was prepared using wood shavings as litter (10 cm thick) and enclosed with a 70 cm-high wooden fence covered with wire mesh. To determine the dissemination of AM residues from the treated group to the untreated group, the experimental area was distributed in an adjacent pen (B.1) and a nearby one (B.2) 30 cm from the treated birds (A), as reported in other studies [15,19]. To ensure proper handling, the study included a control group (C) located in an area separate from the experimental area (see Figure 1).

The study considered the birds breeding from the day of hatching until the day of slaughter (42 days of life). When the birds were 20 days old, those in group A were treated with a commercially available Enrofloxacin formulation. Each bird received a 10 mg·kg^−1^ oral dose every 24 h for 5 days, using an orogastric tube to ensure complete medication intake.

### 2.3. Sampling

For both chemical and microbiological analysis, individual fecal samples were collected from treated and untreated birds by cloacal stimulation in polypropylene tubes. For each experimental group and sampling day, three independent pooled droppings samples (biological replicates; *n* = 3) were collected. Each pool consisted of droppings obtained from different birds within the same group, ensuring that no bird contributed to more than one pool at any given time. Litter samples were obtained using systematic grid sampling, as described in the International Atomic Energy Agency’s [33] report on soil sampling for environmental contaminants. This method involves collecting nine subsamples using a random sampling system that covers the entire corral, which are then pooled and homogenized in a sterile bag to obtain a representative composite sample. Homogenization was achieved by thoroughly vigorous shaking the bag to ensure a uniform distribution of particles and potential contaminants, allowing for an accurate determination of contaminant concentrations. Samples were collected on the third day after the enrofloxacin treatment ended, and then on days 6, 9, 12, 15, and 18 after the treatment.

### 2.4. Solvents, Reagents, Working Solutions and Internal Standards

The extraction and chemical analysis of analytes from the matrices was performed at the Laboratory of Veterinary Pharmacology (FARMAVET) of the Faculty of Veterinary and Animal Sciences at the University of Chile, which is accredited under ISO/IEC 17025:2017 standards [34]. Certified standards with a purity greater than 90% were used. Enrofloxacin (EFX) and ciprofloxacin hydrochloride (CFX), manufactured by Sigma-Aldrich^®^ (St. Louis, MO, USA), were used as the stock solution. Enrofloxacin-D5 (EFX D5) with certified purity (>95%) was used as the internal standard (ES). Samples were extracted using EDTA/McILvaine buffer (0.1 M, pH 4.0 ± 0.1). Two mobile phases were used for chromatographic and extraction analysis. Phase A uses a 2.0 mM ammonium formate solution with 0.16% formic acid in HPLC-grade water. Phase B uses a 2.0 mM ammonium formate solution with 0.16% formic acid in HPLC-grade methanol.

### 2.5. Residue Quantification

EFX and CFX were detected using Ultra Performance Liquid Chromatography ACQUITY UPLC^®^ (Waters™, Milford, MA, USA) coupled to a triple quadrupole mass-mass spectrometer Xevo TQ-S micro (Waters™, Milford, MA, USA). An ACQUITY UPLC REG HSS T3 chromatographic column (1.8 μm, 2.1 × 100 mm; Waters Corporation, Milford, MA, USA) was used. Sample integration was performed using MassLynx™ software version 4.2.

EFX and CFX residues were quantified in litter and manure from treated and untreated broiler chickens using the equation y = a + bx, where “y” is the area ratio, “a” is the y-intercept, “b” is the slope of the line, and “x” is the analyte concentration. A calibration curve was constructed for quantification from antimicrobial-free samples provided by the control group, with a coefficient of determination (R^2^) of ≥0.95.

### 2.6. Microbiological Analysis

The microbiological analysis was performed at the Food Safety Laboratory (INOCUIVET) of the Faculty of Veterinary and Animal Sciences at the University of Chile, which is accredited under ISO 17025 standards.

For the isolation of *E. coli*, samples were obtained by cloacal stimulation, collecting 1 g of fecal sample, which was pre-enriched with 9 mL of Tryptic Soy Broth (BD Difco™, Becton, Dickinson and Company, Sparks, MD, USA). These samples were then homogenized and incubated at 37 °C for 18–24 h. For litter samples, nine subsamples were homogenized, and a final 25 g sample was stored. This sample was pre-enriched in 225 mL of buffered peptone water (Liofil-chem™, Roseto degli Abruzzi, Italy), homogenized for 60 s with a paddle blender, and incubated at 37 °C for 21–24 h. Then, the enriched inoculate was plated in triplicate on MacConkey Agar plates (BD Difco™) and incubated at 37 °C for 18–24 h. Five characteristic *E. coli* colonies were selected from each plate and stored in cryotubes containing Trypticase Soy Broth (TSB) (BD Difco™) and 20% glycerol at −20 °C. For confirmation of the *E. coli* species, DNA extraction from TSA was performed using the boiling method. A sample from that agar was collected with an inoculation loop and then suspended in sterile physiological saline in a 1.5 mL polypropylene tube. This solution was homogenized and centrifuged for 2 min. The supernatant was removed, and 1 mL of sterile physiological saline was added to each tube to wash and re-homogenize. Samples were boiled at 100 °C in a Thermoblock (ACCUBLOCK™, Labnet International, Inc., Edison, NJ, USA) for 10 min. Finally, the tubes were centrifuged for 10 min in a Model D1524R micro-centrifuge (DLAB, Scientific Co., Ltd., Beijing, China), and 400 μL of the supernatant was stored in Eppendorf tubes at −20 °C. The *uspA* gene was identified using conventional polymerase chain reaction (PCR) to confirm the bacterial species [35].

### 2.7. Antimicrobial Resistance

The antimicrobial susceptibility of *E. coli* isolates was determined using the in vitro Kirby-Bauer disk diffusion method [36]. First, the inoculum was prepared from colonies grown in a pure culture incubated for 16–18 h at 37 °C on MacConkey agar. The optical density (OD) was then adjusted spectrophotometrically to an OD 600 nm of between 0.08 and 0.13 (equivalent to a 0.5 McFarland standard). The inoculum was transferred to Müller-Hinton Agar (Sigma-Aldrich) using a sterile swab until the agar surface was completely covered. The plate was allowed to dry for 3–5 min, and then an enrofloxacin (5 μg) and ciprofloxacin (5 μg) sensidisk (OXOID^®^, Basingstoke, UK) was placed on the surface of the inoculated agar. The plates were incubated with the samples inverted at 35 °C for 18 h. The reference strain *E. coli* ATCC 25922 was used as a quality control. Antimicrobial susceptibility results were read and interpreted by measuring the inhibition zones in millimeters, following the guidelines indicated in the document “Clinical and Performance Standards for Antimicrobial Disk and Dilution Susceptibility Test for Bacteria Isolated from Animals of the Laboratory Standards Institute” [37].

### 2.8. Statistical Analysis

The recommendations of the European Medicines Agency [38] were followed to determine the persistence of EFX and CFX in the litter matrix, identifying the day on which the concentration of each antimicrobial was equal to or lower than the limit of quantification (LOQ). Microsoft^®^ Excel Microsoft 365 (Version 2408, Microsoft Corporation, Redmond, WA, USA) was used to transform the concentrations to a logarithmic scale, and a linear regression analysis was performed, using antimicrobial residue concentrations as the dependent variable and time as the quantitative predictor variable. A 95% confidence level was used to determine the time to depletion, and the coefficient of determination (R^2^) was estimated to evaluate the linear model.

To assess the dissemination of EFX and CFX analytes in the production environment, data normality was first assessed with the modified Shapiro-Wilks test and homoscedasticity was tested with Levene’s test. Since these criteria were not met, the nonparametric Kruskal–Wallis test was performed, using a *p*-value < 0.05 and a 95% confidence interval, to determine statistically significant differences between the median concentrations of EFX and CFX of the study groups. Dunn’s test was applied to evaluate the difference in median concentrations between groups. Additionally, a descriptive analysis of the resistance profiles of *E. coli* to fluoroquinolones was conducted. Kruskal–Wallis test was performed to evaluate the difference in *E. coli* resistance rates between experimental groups, independently on each sampling day. To estimate the strength of association between the variables, a Pearson correlation analysis was performed following Akoglu’s criteria [39].

## 3. Results

### 3.1. Persistence of EFX and CFX Residues in Non-Edible By-Products After Treatment

The average concentrations of EFX detected in the litter and droppings of the treated group (A) 3 days post-treatment were 20,968 µg kg^−1^ and 884.8 µg kg^−1^, respectively. A gradual decrease was observed throughout the production cycle, with concentrations exceeding the limit of quantification (LOQ > 5 µg kg^−1^) for up to 18 days post-treatment. CFX concentrations above the LOQ were detected in droppings up to day 12 post-treatment, while in litter, concentrations of 609.9 µg kg^−1^ were detected 18 days post-treatment. Concentration levels varied significantly between sampling days in group A (Kruskal–Wallis test; *p* < 0.05), with higher concentrations observed on the first sampling days, followed by a marked decrease on subsequent days. Dunn’s post hoc test with Bonferroni correction showed that day 3 post-treatment had a significantly higher concentration than day 15, while days 6, 9, 12, and 18 showed intermediate values with no significant differences between them (Table 1).

For the control group (C), no concentrations were detected for both litter and droppings.

### 3.2. Persistence and Dissemination of Antimicrobial Residues in the Poultry Environment

**Figure 2:**
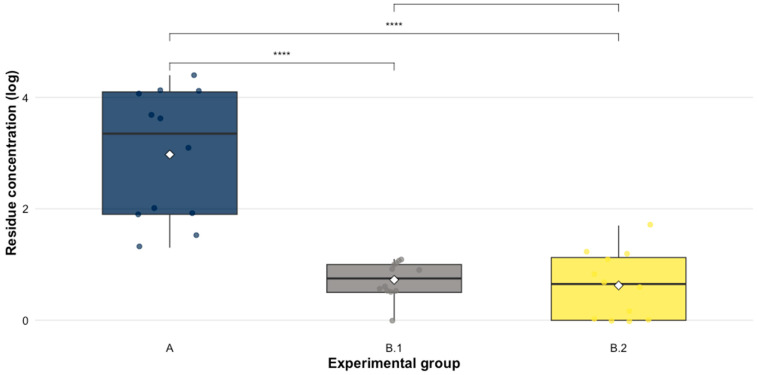
Comparison of detected residue concentrations in natural logarithmic scale of EFX + CFX between experimental groups post-treatment (**** significant difference).

To determine the spread of EFX and CFX residues within the environment to the untreated groups (B.1 and B.2), the concentration of these analytes was quantified in litter and droppings (Table 2). The non-parametric Kruskal–Wallis test determined significant differences in residue concentrations between the experimental groups (χ^2^ = 22.82, *p*-value < 0.005). Dunn’s test with Bonferroni correction indicated that group A had significantly higher concentrations compared to groups B.1 (*p*-value = 1.8 × 10^−4^) and B.2 (*p*-value = 6.4 × 10^−5^), while no significant differences were observed between B.1 and B.2, demonstrating a marked effect of the treatment on excretion and accumulation residue in litter of treated birds (see Figure 2).

### 3.3. Effect of Residues of EFX and CFX on the Antimicrobial Resistance of E. coli

The susceptibility of *E. coli* isolates obtained from litter and droppings to EFX and CFX was evaluated. The treated group (A) exhibited the highest mean resistance rates (73%), while the untreated groups B.1 (24%) and B.2 (13%). The control group showed a resistance rate of 3.3%.

Significant differences in *E. coli* resistance rates were detected between experimental groups independently on each sampling day (Kruskal–Wallis, *p* < 0.05). Resistance rates varied among experimental groups depending on the sampling day and matrix. In the litter matrix, Group A showed a consistently higher resistance rate on all sampling days compared to Groups B.1 and B.2. Significant differences between Group A and the untreated groups were detected on days 3, 9, and 12 post-treatment (Dunn test with Bonferroni correction, *p* < 0.05), while no significant differences were observed on days 6, 15, and 18 (*p* > 0.05) (Table 3). In droppings, Group A also exhibited higher resistance rates, with significant group-level differences observed on days 3 and 12 post-treatment (Dunn test with Bonferroni correction, *p* < 0.05), while no significant differences were detected on days 6, 9, 15, and 18 despite the numerically higher mean values in Group A (Table 4).

The relationships between experimental groups, antimicrobial residue concentration, days post-treatment, resistance rate, matrix, and analyte were evaluated using Spearman’s correlation. The results showed a strong positive correlation between treatment and detected residue concentration. A significant positive association was observed between the resistance rate of *E. coli* and quantifiable residue concentrations when assessed using Spearman’s correlation (ρ = 0.53, *p* < 0.001).

**Figure 3:**
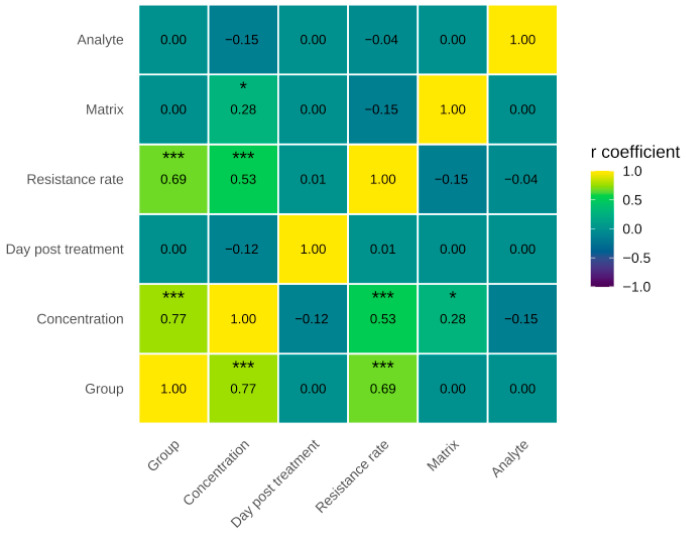
Heatmap where the associations between the variables are represented: experimental groups, the concentration of antimicrobial residues, the days after treatment, the resistance rate, the matrix and the analyte using Spearman correlation (*** *p* < 0.001, * *p* < 0.05).

Furthermore, a moderate positive correlation between residue concentration and *E. coli* resistance rate supports the hypothesis that the persistence of antimicrobial residues in the environment contributes to the selection of resistant bacterial populations. On the other hand, weak correlations were observed between residue concentration and matrix, as well as between concentration and analyte, indicating that, although residue levels varied slightly depending on the environmental matrix, this factor was not a major determinant of *E. coli* resistance to fluoroquinolones (see Figure 3).

## 4. Discussion

This study evaluates the concentration of EFX and CFX residues in litter and droppings after a treatment with a commercial formulation of Enrofloxacin. In group A (treated animals), the concentrations initially detected on the third day post-treatment gradually decreased over time. Persistence in litter and droppings, with concentrations above the limit of quantification (LOQ > 5 µg kg^−1^) was measured up to 18 days post-treatment. Similar results were obtained by Swinkels et al. [25], who found that EFX concentrations in feces far exceeded the range of selective minimum concentrations (MSCs) after treatment and remained within that range for up to 26 days post-treatment. Studies have quantified fluoroquinolone residues in litter, finding concentrations of up to 24,307 µg kg^−1^ for EFX and CFX combined [16], coinciding with the concentrations obtained in our study. The persistence of fluoroquinolones can be attributed to the functional groups that comprise the structure, which determine the affinity for various surfaces and facilitate adsorption onto organic matrices [40]. Alvarez-Esmorís et al. [41] investigated the adsorption process of EFX in calcined and uncalcined agricultural soils to assess the impact of organic matter on this process. Their results indicated that organic matter plays a fundamental role in enhancing the adsorption process of EFX.

An interesting finding regarding the concentrations of EFX and CFX detected in the litter of group A was that these concentrations increased at the end of the production cycle (18 days post-treatment). This could be explained by the fact that the litter contains various components, such as excrement and feathers, which can contain residues of the antimicrobial used in the treatment of the birds and act as a source of litter contamination. This was reported in a study by San Martín et al. [42], which evaluated the depletion of EFX and CFX in edible tissues and feathers of chickens. The results showed that concentrations of EFX combined with CFX reached 1279 µg kg^−1^ on the third day post-treatment, decreasing to 33.1 µg kg^−1^ by the ninth day post-treatment. The phenomenon of antimicrobial recirculation must be considered when examining the variation in concentrations obtained in this experiment. It has been documented that the chemical stability of antimicrobials, such as EFX, combined with the coprophagic behavior of birds, can lead to drug recirculation and, simultaneously, persistence [25]. Furthermore, it has been described that the main route of litter contamination with antimicrobial residues is through the excretion of residues in the droppings of birds treated with the drug. Several authors have conducted studies detecting fluoroquinolones in poultry droppings after administration of therapeutic doses [43]. This family of antimicrobials has been found in concentrations up to 41,800 µg kg^−1^ [44], and it has been possible to quantify it up to day 18 post-treatment in this matrix [15].

The results of this study showed that EFX exhibited limited dissemination to the untreated groups (B.1 and B.2); however, this dispersion was greater compared to CFX. The metabolism and excretion of EFX in broiler chicken feces following oral administration of the antimicrobial have been described. It has been reported that approximately 74% of the oral dose is excreted as the parent compound (EFX) and 25% as the main metabolite, CFX [45]. Although CFX has very similar physicochemical properties to the parent compound, it is important to consider that the metabolism of EFX to CFX involves the dedistillation of the ethyl group on the piperazine ring, which gives CFX better water solubility than EFX [22]. Therefore, it could be expected that the adsorption of CFX onto broiler litter will be lower, and this analyte will be more susceptible to environmental degradation.

Similar results have been observed in other studies evaluating the dissemination of oxytetracycline, sulfachloropyridazine, and tylosin [19,20,46]. These studies have demonstrated the persistence and dissemination of residues in the agricultural environment following treatment. The concentrations of EFX and CFX indicate that animals in groups B.1 and B.2 were exposed to antimicrobial residues, likely through the dispersal of antimicrobial-containing dust from the treatment group, generated by the birds’ movement in the litter during dust baths [47].

An *E. coli* resistance rate of 73% was obtained in group A, 24% in group B.1, 13% in group B.2 and 3.3% in the control group. Resistance throughout the experimental period showed a marked decrease in resistance until day 9 post-treatment, suggesting an initial loss of resistance that may be associated with reduced selective pressure or depletion of the antimicrobial in the environment. However, from day 12, the values show a partial recovery, suggesting a dynamic adaptation of the microbial population. This could be explained by mutations in the *gyrA* gene, which has been reported as one of the main factors contributing to high resistance to quinolones [48].

The results showed a positive relationship between treatment and resistance rate. This is consistent with findings reported by other authors who examined how the recirculation of persistent antimicrobials in poultry production systems can prolong the selection of antimicrobial resistance to *E. coli* in broilers, finding that the group treated with EFX showed 100% phenotypic resistance in *E. coli* at all sampling points [25]. It has been suggested that the selective pressure for the development of antimicrobial resistance intensifies in the presence of antimicrobials, as evidenced by the fact that even under inhibitory and subinhibitory concentrations of these compounds, bacteria with the ability to survive and proliferate are selected [49].

The strong positive correlation between treatment and the concentration of detected residues, as well as between treatment and the fluoroquinolone resistance rate of *E. coli*, indicates that exposure to the treatment increased both the presence of residues and the levels of bacterial resistance. Likewise, the moderate positive correlation between residue concentration and resistance rate (ρ = 0.53) supports the hypothesis that the persistence of antibiotics in the environment promotes a tendency to the selection of resistant populations. This finding is consistent with previous research indicating that the use of antimicrobials in production systems exerts selective pressure on commensal bacteria, favoring the proliferation of resistant strains [19,50]. However, the concentration of antimicrobial residues reached in the litter may have been too low for the selection of fluoroquinoline-resistant *E. coli* isolates due to the mutant selection window (MSW), where the lower limit of the MSW represents the lowest concentration of an antibiotic at which the proliferation of wild-type bacteria is suppressed, and below this concentration the mutants do not have a selective advantage over WT *E. coli* [51].

This study represents a first step in characterizing the behavior of fluoroquinolones in intensive poultry production systems under controlled conditions and evaluating the effect on the development of antimicrobial resistance. Its main contribution lies in describing the role of byproducts, such as litter and droppings, as a source of EFX residues, highlighting the presence of CFX, a high-priority antimicrobial of critical importance to human health, within the context of poultry production. These findings contribute to understanding the significant implications of antimicrobial use in the animal industry for other areas, underscoring the need to continue this line of research within the framework of the One Health approach.

## 5. Conclusions

This study provides experimental evidence on the persistence and limited dissemination of EFX and its metabolite, CFX, under controlled conditions, suggesting that antimicrobial residues can spread in the production environment. This work highlights the importance of litter and droppings as significant reservoirs of antimicrobial residues and critical components in antimicrobial resistance, contributing to the maintenance of selective pressure in poultry facilities. These results reinforce the relationship between antimicrobial use, the presence of residues in the environment and antimicrobial resistance, reinforcing the importance of litter and byproduct management from a One Health perspective.

These findings underscore the need to improve the management of non-edible by-products and environmental monitoring strategies to mitigate the spread of antimicrobials in poultry production systems.

From a One Health perspective, addressing the environmental dimension of antimicrobial use is essential to prevent the cross-sectoral transmission of antimicrobial resistance and to safeguard public and animal health.

## Figures and Tables

**Table 1:** Concentrations of EFX and CFX residues detected after treatment in litter and droppings of the treated group.

Enrofloxacin and Ciprofloxacin Residues Concentrations (µg·kg^−1^ ww)							
Residue	Matrix	Day 3	Day 6	Day 9	Day 12	Day 15	Day 18
EFX	Litter	20,968 ^a^	11,362 ^ab^	12,012 ^ab^	10,035 ^ab^	3366 ^b^	4997 ^ab^
	Droppings	885 ^a^	82 ^ab^	61 ^ab^	67 ^ab^	26 ^ab^	19 ^b^
CFX	Litter	4558 ^a^	2135 ^ab^	1684 ^ab^	1300 ^ab^	401 ^b^	610 ^ab^
	Droppings	423 ^a^	24 ^ab^	20 ^ab^	14 ^ab^	<LOD ^b^ *	<LOD ^b^

Different superscript letters within each row indicate statistically significant differences among days post-treatment (Dunn’s test with Bonferroni correction *p* < 0.05). * Limit of detection: minimum concentration of a substance that an analytical method can reliably identify (LOD = 2.5 µg kg^−1^). Limit of quantification: lowest concentration of a substance that can be reliably and reproducibly measured (LOQ = 5 µg kg^−1^).

**Table 2:** Concentrations of EFX and CFX residues detected on sampling days after treatment in litter and droppings of the untreated group.

	Enrofloxacin and Ciprofloxacin Residues Concentrations (µg·kg^−1^ ww)							
Group	Residue	Matrix	Day 3	Day 6	Day 9	Day 12	Day 15	Day 18
B.1	EFX	Litter	14.00	7.86	8.08	11.05	10.62	13.86
		Droppings	<LOD *	<LOD	<LOD	<LOD	<LOD	<LOD
	CFX	Litter	<LOD	<LOD	<LOD	<LOD	<LOD	<LOD
		Droppings	<LOD	<LOD	<LOD	<LOD	<LOD	<LOD
B.2	EFX	Litter	5.83	5.44	51.00	14.75	16.81	13.01
		Droppings	<LOD	<LOD	<LOD	<LOD	<LOD	<LOD
	CFX	Litter	<LOD	<LOD	<LOD	<LOD	<LOD	<LOD
		Droppings	<LOD	<LOD	<LOD	<LOD	<LOD	<LOD

* Limit of detection: minimum concentration of a substance that an analytical method can reliably identify (LOD = 2.5 µg kg^−1^). Limit of quantification: lowest concentration of a substance that can be reliably and reproducibly measured (LOQ = 5 µg kg^−1^).

**Table 3:** Resistance rate of *E. coli* by group and sampling day post-treatment in litter.

Day Post-Treatment	Group A	Group B.1	Group B.2
3	1.00 ± 0.00 ^a^	0.00 ± 0.00 ^b^	0.00 ± 0.00 ^b^
6	0.67 ± 0.33 ^a^	0.00 ± 0.00 ^a^	0.00 ± 0.00 ^a^
9	1.00 ± 0.00 ^a^	0.08 ± 0.08 ^ab^	0.00 ± 0.00 ^b^
12	1.00 ± 0.00 ^a^	0.00 ± 0.00 ^b^	0.00 ± 0.00 ^b^
15	0.88 ± 0.12 ^a^	0.00 ± 0.00 ^a^	0.00 ± 0.00 ^b^
18	1.00 ± 0.00 ^a^	1.00 ± 0.00 ^a^	0.00 ± 0.00 ^b^

Note: Values are expressed as mean ± standard error (SE). Different letters within the same row indicate statistically significant differences between groups for the same day (Kruskal–Wallis followed by Dunn post hoc test with Bonferroni correction, *p* < 0.05).

**Table 4:** Resistance rate of *E. coli* by group and sampling day post-treatment in droppings.

Day Post Treatment	Group A	Group B.1	Group B.2
3	1.00 ± 0.00 ^a^	0.00 ± 0.00 ^b^	0.20 ± 0.20 ^ab^
6	0.93 ± 0.07 ^a^	0.27 ± 0.13 ^a^	0.60 ± 0.20 ^a^
9	0.00 ± 0.00 ^a^	0.00 ± 0.00 ^a^	0.07 ± 0.07 ^a^
12	0.50 ± 0.17 ^a^	0.13 ± 0.07 ^ab^	0.00 ± 0.00 ^b^
15	0.63 ± 0.09 ^a^	0.60 ± 0.12 ^a^	0.00 ± 0.00 ^a^
18	0.73 ± 0.18 ^a^	0.50 ± 0.17 ^a^	0.00 ± 0.00 ^a^

Note: Values are expressed as mean ± standard error (SE). Different letters within the same row indicate statistically significant differences between groups for the same day (Kruskal–Wallis followed by Dunn post hoc test with Bonferroni correction, *p* < 0.05).

## Data Availability

The data presented in this study are available in the article.
